# Investigating the role of imaging factors in the variability of CT‐based texture analysis metrics

**DOI:** 10.1002/acm2.14192

**Published:** 2023-11-14

**Authors:** Bino Abel Varghese, Steven Yong Cen, Kristin Jensen, Joshua Levy, Hilde Kjernlie Andersen, Anselm Schulz, Xiaomeng Lei, Vinay Anant Duddalwar, David John Goodenough

**Affiliations:** ^1^ Keck Medical Center Department of Radiology University of Southern California Los Angeles California USA; ^2^ Department of Physics and Computational Radiology Oslo Norway; ^3^ The Phantom Laboratory Greenwich New York USA; ^4^ Department of Radiology and Nuclear Medicine Oslo University Hospital Oslo Norway; ^5^ Department of Radiology George Washington University Washington District of Columbia USA

**Keywords:** computed tomography, imaging, phantom study, quality assurance, radiomics

## Abstract

**Objective:**

This study assesses the robustness of first‐order radiomic texture features namely interquartile range (IQR), coefficient of variation (CV) and standard deviation (SD) derived from computed tomography (CT) images by varying dose, reconstruction algorithms and slice thickness using scans of a uniform water phantom, a commercial anthropomorphic liver phantom, and a human liver in‐vivo.

**Materials and Methods:**

Scans were acquired on a 16 cm detector GE Revolution Apex Edition CT scanner with variations across three different nominal slice thicknesses: 0.625, 1.25, and 2.5 mm, three different dose levels: CTDIvol of 13.86 mGy for the standard dose, 40% reduced dose and 60% reduced dose and two different reconstruction algorithms: a deep learning image reconstruction (DLIR‐high) algorithm and a hybrid iterative reconstruction (IR) algorithm ASiR‐V50% (AV50) were explored, varying one at a time. To assess the effect of non‐linear modifications of images by AV50 and DLIR‐high, images of the water phantom were also reconstructed using filtered back projection (FBP). Quantitative measures of IQR, CV and SD were extracted from twelve pre‐selected, circular (1 cm diameter) regions of interest (ROIs) capturing different texture patterns across all scans.

**Results:**

Across all scans, imaging, and reconstruction settings, CV, IQR and SD were observed to increase with reduction in dose and slice thickness. An exception to this observation was found when using FBP reconstruction. Lower values of CV, IQR and SD were observed in DLIR‐high reconstructions compared to AV50 and FBP. The Poisson statistics were more stringently noted in FBP than DLIR‐high and AV50, due to the non‐linear nature of the latter two algorithms.

**Conclusion:**

Variation in image noise due to dose reduction algorithms, tube current, and slice thickness show a consistent trend across phantom and patient scans. Prospective evaluation across multiple centers, scanners and imaging protocols is needed for establishing quality assurance standards of radiomics.

## 1 INTRODUCTION

Advancements in imaging technology, computational power and artificial intelligence (AI) have ushered in a powerful approach of image characterization called Radiomics.^1^, ^2^ Defined as the high throughput extraction of mineable quantitative metrics from routine medical images, radiomics has found applications in a variety of diagnostic, prognostic, treatment evaluation applications in variety of diseases most popularly cancer.^2^, ^3^ Radiomic metrics capturing tumor shape, nonuniform grayscale appearance (texture) which are difficult to assess visually have been reported to provide information regarding tumor diagnosis, prognosis, and treatment response. However, despite various benefits within the clinical workflow such as objective whole tumor assessment at no additional imaging cost and longitudinal disease monitoring, current limitations with the standardization of the method may reduce its reliability, particularly in multicenter studies.^4^, ^5^, ^6^, ^7^

Even within CT there is a great effort towards imaging using lower doses, particularly for the pediatric population and also for special indications such as low dose screening studies in the lung and the colon. For example, the automatic exposure control adapts scanner output to patient size.^8^ Advanced image reconstruction techniques using iterative reconstruction and deep learning reduce noise and may simultaneously maintain resolution.^9^, ^10^, ^11^, ^12^, ^13^, ^14^ More efficient CT detector systems (photon counting CT) that require fewer photons and less dose are underway.^15^ While these advancements substantially reduce the dose to the patients for comparable diagnostic value, the effect these dose improvement technologies on quantitative assessment of the same images is not well understood.^16^, ^17^, ^18^ While Li et al reported on the effect of newer reconstruction techniques on volumetric assessments assessed using a anthropomorphic liver phantom, the effects on radiomic metrics extracted from such CT images are poorly studied.^19^ While machine learning (ML), AI and statistical solutions are being developed to harmonize / standardize radiomics metrics or the images from where these metrics are extracted, studies exploring the dependencies and relationships that exist between the radiomics metric and various CT techniques are highly warranted.^20^

Without standardization of the radiomics process across multiple centers, a valid translation of radiomics to clinical practice is a challenge.^5^, ^21^, ^22^, ^23^ Establishing reliable associations between the CT‐based texture analysis (CTTA) metrics and imaging variables across different vendors and acquisition protocols may provide the quality assurance required for clinical radiomics. Several phantom studies in the literature reported on the evaluation of the variability of CT‐based texture features under different CT scanner parameters and vendors.^6^, ^24^, ^25^, ^26^ For certain types of features, applying image‐filtering techniques improves their reliability, while in other cases applying a voxel size normalization helps to increase robustness of certain features across different manufactured CT scanners. Considering the wide variability in image acquisition, reconstruction, processing options and the lack of standardization of the radiomics process, additional studies comprehensively assessing the relationship between imaging parameters and radiomics metrics are warranted. In addition, such studies should also evaluate the feasibility of establishing some quality assessment thresholds prior to conducting radiomics analysis using both handcrafted features and deep learning‐based features.^20^

In this study, the robustness of some key first‐order texture features namely interquartile range (IQR), coefficient of variation (CV) and standard deviation (SD) derived from computed tomography (CT) images are assessed by varying dose, reconstruction algorithms and slice thickness using scans of a uniform water phantom, anthropomorphic liver phantom, and a human liver in vivo. The rationale for choosing these simple first‐order metrics is to better understand the variation of these easy‐to‐understand texture metrics with the aforementioned image acquisition parameters. At a fundamental level assessing the variation of the SD, CV show the extent of variability in relation to the mean of the region of interest and IQR shows measure of statistical dispersion, specifically difference between the 75th and 25th percentiles of the data. Taken together, these three quantities help to gain a good understanding of the dispersion of values from a mean within a given region of interest. The aim is to determine the difference in these first‐order radiomic texture features, all of which capture the dispersion of values from a mean, within a region of interest due to change in imaging variables such as dose, slice thickness and choice of reconstruction algorithm versus due to change in underlying structure. This exercise will help establish minimum thresholds of image quality for conducting radiomics analysis, whose goal must be to capture the difference due to underlying pathology and not imaging related changes in radiomic metric values.

## 2 METHODS

### 2.1 Phantom design

In the study, to evaluate the reliability of the CTTA metrics, we conducted a series of imaging experiments using a large water phantom and an anthropomorphic liver phantom. The water phantom is a PMMA cylinder, 32 cm in diameter, 12 cm in length and filled with water, provided by Canon for CT scanner calibration. The anthropomorphic liver phantom^27^ is a simplified representation of a human torso with a 25 cm x 35 cm oval shape and a length of 15 cm. The phantom body is cast from RANDO® tissue simulating material and contains simplified liver and bone structures. The liver is cast out of a higher density tissue material and the vertebrae and ribs are cast from simulated bone materials. The phantom has seven 5 cm diameter holes for holding insert uniform rods. Three of the rods are cast from RANDO® material and placed in the phantom body and four of the rods cast from liver material are placed within the liver.

### 2.2 In vivo CT human liver

A male patient (68 years old) with inoperable metastases from colorectal cancer receiving palliative treatment was scanned with the same protocol as the phantoms. Written informed consent was obtained.

### 2.3 CT imaging and reconstruction

All CT scans were acquired using a 16 cm detector GE Revolution Apex Edition CT scanner (GE Healthcare, Waukesha, Wisconsin) (Table 1). Variations across three different nominal slice thicknesses: 0.625, 1.25, and 2.5 mm, three different dose levels: the standard dose (CTDIvol of 13.86 mGy), 40% reduced dose and 60% reduced dose and two different reconstruction algorithms: a deep learning image reconstruction (DLIR‐high, TrueFidelity, GE Healthcare) algorithm and a hybrid iterative reconstruction (IR) algorithm ASiR‐V50% (AV50) were explored, varying one at a time. Dose modulation was used to get similar image quality in phantoms and the patient. Noise index was set to 29 (using 0.625 mm slice thickness and ASiR‐V0%) and for the dose reduced scans noise index was adjusted to get CTDIvol reductions of 40% and 60%. To assess the effect of non‐linear modifications of images by AV50 and DLIR‐high, images of a large water phantom were also reconstructed using FBP algorithm.

**TABLE 1:** CT acquisition parameters.

	Water phantom	Liver phantom	In‐vivo liver scan
	Helical		
**Tube voltage (kVp)**	120		
**Tube current (mA)**	222	214	242
**Rot.time (s)**	1		
**Pitch**	0.508		
**DFOV (mm)**	430	430	400
**SFOV**	Body filter		
**Collimation (mm)**	80		
**Kernel**	Standard		
**Noise index**	29		
**CTDIvol (mGy)**	14.7	11.4	13.9

### 2.4 Region of interest segmentation

**FIGURE 1:**
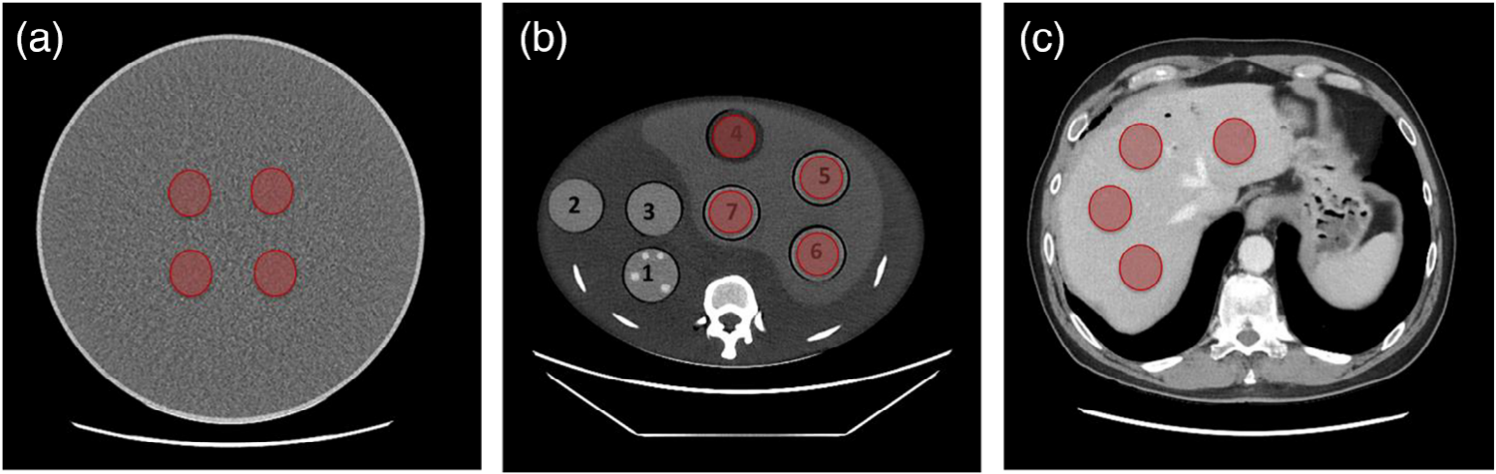
Regions of interest (highlighted in red) across the three imaging scenarios: (1) large water phantom; (2) commercial anthropomorphic liver phantom (CCT288, Phantom Lab, NY); and (3) an in vivo human liver scan.

The region of interest (ROI) delineation was performed using a manual segmentation technique. On the large water phantom four circular ROIs ∼1 cm diameter each were manually segmented over five adjacent CT slices (Figure 1_1). On the anthropomorphic liver phantom four Circular ROIs ∼1 cm diameter each were manually segmented over five adjacent CT slices encompassing the uniform rods that pass through the holes of the liver region (Figure 1_2). Similarly, in the in‐vivo liver CT scans four Circular ROIs ∼1 cm diameter each were manually segmented over five adjacent CT slices where apparently normal liver tissue was present (Figure 1_3). Regions with obvious blood vessels were avoided. In all cases, segmentation was performed using an open‐source software package CapTk (version‐1.8.1).^28^ Care was taken to not include edges within the mostly uniform ROIs.

### 2.5 Radiomics extraction

From the three types of ROIs, that is, those extracted from the phantoms and the patient, average of three first‐order statistical texture metrics namely SD, CV, and IQR, respectively, were extracted to study the noise characteristics of the various imaging settings. These three first‐order statistical measures of texture were extracted in compliance with the Image Biomarker Standardization Initiative (IBSI),^5^ using the publicly available Cancer Imaging Phenomics Toolkit (CaPTk, https://www.cbica.upenn.edu/captk).^28^

### 2.6 Statistical analysis

From the images acquired for each setting, across the three different imaging scenarios, that is, the large water phantom, the anthropomorphic liver phantom, and the in‐vivo human liver CT scan, four ROIs (Figure 1) were manually segmented and SD, CV, and IQR were extracted from them. The extracted values were plotted using bar plots and used to visualize the trend of these variables due to changing slice thickness, dose and reconstruction algorithms. The data normality was examined by histogram and D'Agostino's *K* ^2^ test. If data were not normally distributed, Wilcoxon rank score transformation was used. Generalized linear models were used to test the trend effect. Residual plots were used to inspect the model integrity. SAS 9.4 (SAS Institute, Cary, North Carolina) was used for all data analyses.

## 3 RESULTS

Our results indicate that the values of SD, CV and IQR of an ROI are dependent on the scanning and reconstruction settings. While these metrics are part of first‐order texture metrics to assess ROI heterogeneity as part of a radiomics panel, assessing these quantities on structureless ROIs such as water, will help differentiate the changes in these values due to imaging noise related changes compared to underlying structural changes. The trends of these metrics across three different imaging settings and scan types are reported.

### 3.1 Water phantom: Effect of slice thickness, dose levels and reconstruction on first order radiomic features: SD, CV, and IQR

**FIGURE 2:**
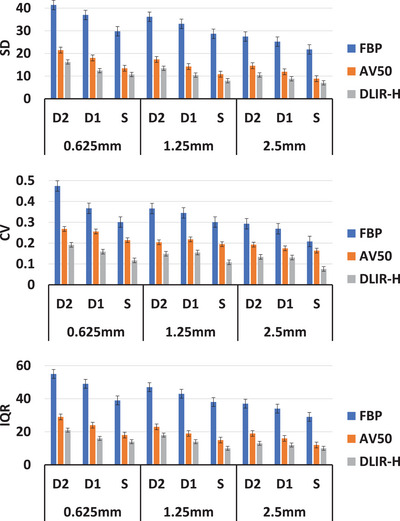
Distribution of SD (top) CV (middle) and IQR (bottom) extracted from the ROIs of a large water phantom, with changes in slice thickness (0.625 mm vs. 1.25 mm vs. 2.5 mm), dose (standard dose (S), 40% of S (D1) and 60% of S (D2)) and reconstruction algorithm (FBP vs. AV50 vs. DLIR‐high).

In general, SD, CV, and IQR improve (decrease) as slice thickness increases (coarse *z*‐axis resolution) (Figure 2). These quantities also improve as dose increases (Figure 2). The relative improvements of SD, CV, and IQR are also higher when using DLIR‐high compared to AV50 (Figure 2). This means for the same changes in dose, that is, D2 to D1 to S, the associated improvements in SD, CV, and IQR are comparatively higher when using DLIR‐high than AV‐50. All trend tests were statistically significant (*p* < 0.01) The details of slopes and statistical trend tests were shown in Table 2.

**TABLE 2:** Details of slopes and statistical trend tests.

	Coefficient of variation			Interquartile range			Standard deviation		
Water phantom	Slope	±SD	*p*‐value	Slope	±SD	*p*‐value	Slope	±SD	*p*‐value
Reducing dose^*^	12.92	3.83	<0.01	13.88	3.79	<0.01	16.08	3.73	<0.01
Changing AV50 to DLIR‐high	−29.54	5.97	<0.01	−51.48	4.60	<0.01	−41.16	5.39	<0.01
Increasing slice thickness^*^	−15.15	3.76	<0.01	−20.50	3.54	<0.01	−17.85	3.67	<0.01

**Liver phantom**	**Slope**	**±SD**	***p*‐value**	**Slope**	**±SD**	***p*‐value**	**Slope**	**±SD**	***p*‐value**
Reducing dose^*^	17.77	4.29	<0.01	17.67	4.74	<0.01	18.97	4.96	<0.01
Changing AV50 to DLIR‐high	−40.62	6.69	<0.01	−65.53	5.75	<0.01	−48.57	7.17	<0.01
Increasing slice thickness^*^	−20.83	4.21	<0.01	−26.10	4.43	<0.01	−21.06	4.88	<0.01

**Patient scan**	**Slope**	**±SD**	***p*‐value**	**Slope**	**±SD**	***p*‐value**	**Slope**	**±SD**	***p*‐value**
Reducing dose^*^	19.48	4.80	<0.01	19.51	5.24	<0.01	21.11	5.46	<0.01
Changing AV50 to DLIR‐high	−44.53	7.48	<0.01	−72.36	6.36	<0.01	−54.02	7.88	<0.01
Increasing slice thickness^*^	−22.84	4.71	<0.01	−28.81	4.89	<0.01	−23.43	5.37	<0.01

* Three levels were considered.

Abbreviations: SD, standard deviation; slope measured as beta value.

**FIGURE 3:**
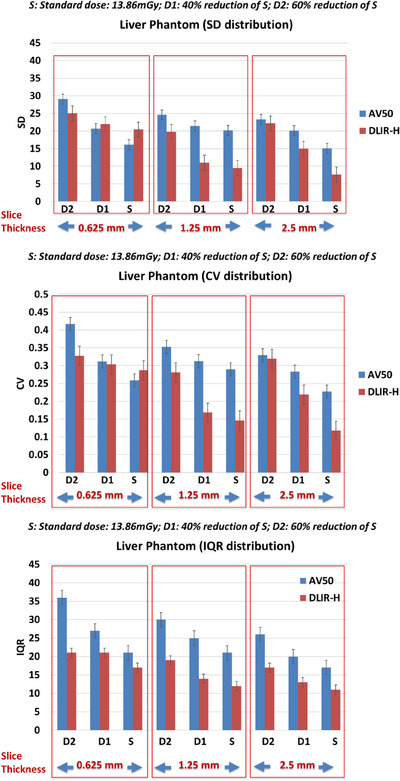
Distribution of SD (top) CV (middle) and IQR (bottom) extracted from the ROIs of an anthropomorphic liver phantom, with changes in slice thickness (0.625 mm vs. 1.25 mm vs. 2.5 mm), dose (standard dose (S), 40% of S (D1) and 60% of S (D2)) and reconstruction algorithm (AV50 vs. DLIR‐high).

Anthropomorphic liver phantom: Effect of slice thickness, dose levels and reconstruction on first order radiomic features: SD, CV, and IQR: In general, as previously observed in the water phantom, SD, CV, and IQR improve as slice thickness increases (coarse *z*‐axis resolution) (Figure 3). These quantities also improve as dose increases (Figure 3). At slice thicknesses of 1.25 mm and 2.5 mm, the relative improvement of SD, CV, and IQR are also higher when using DLIR‐high compared to AV50 (Figure 3). This means that at slice thicknesses of 1.25 mm and 2.5 mm for the same changes in dose, that is, D2 to D1 to S, the associated improvements in SD, CV and IQR are comparatively higher when using DLIR‐high than AV‐50. However, at the lowest resolution of 0.625 mm, this is not observed, possibly due to increased noise associated with small slice thickness. While for the same change in dose, the improvement of IQR is higher when using DLIR‐high compared to AV50, the improvement of SD and CV are lower when using DLIR‐high compared to AV50. All trend tests were statistically significant (*p* < 0.01) The details of slopes and statistical trend tests were shown in Table 2.

### 3.2 In‐vivo human liver CT scans

**FIGURE 4:**
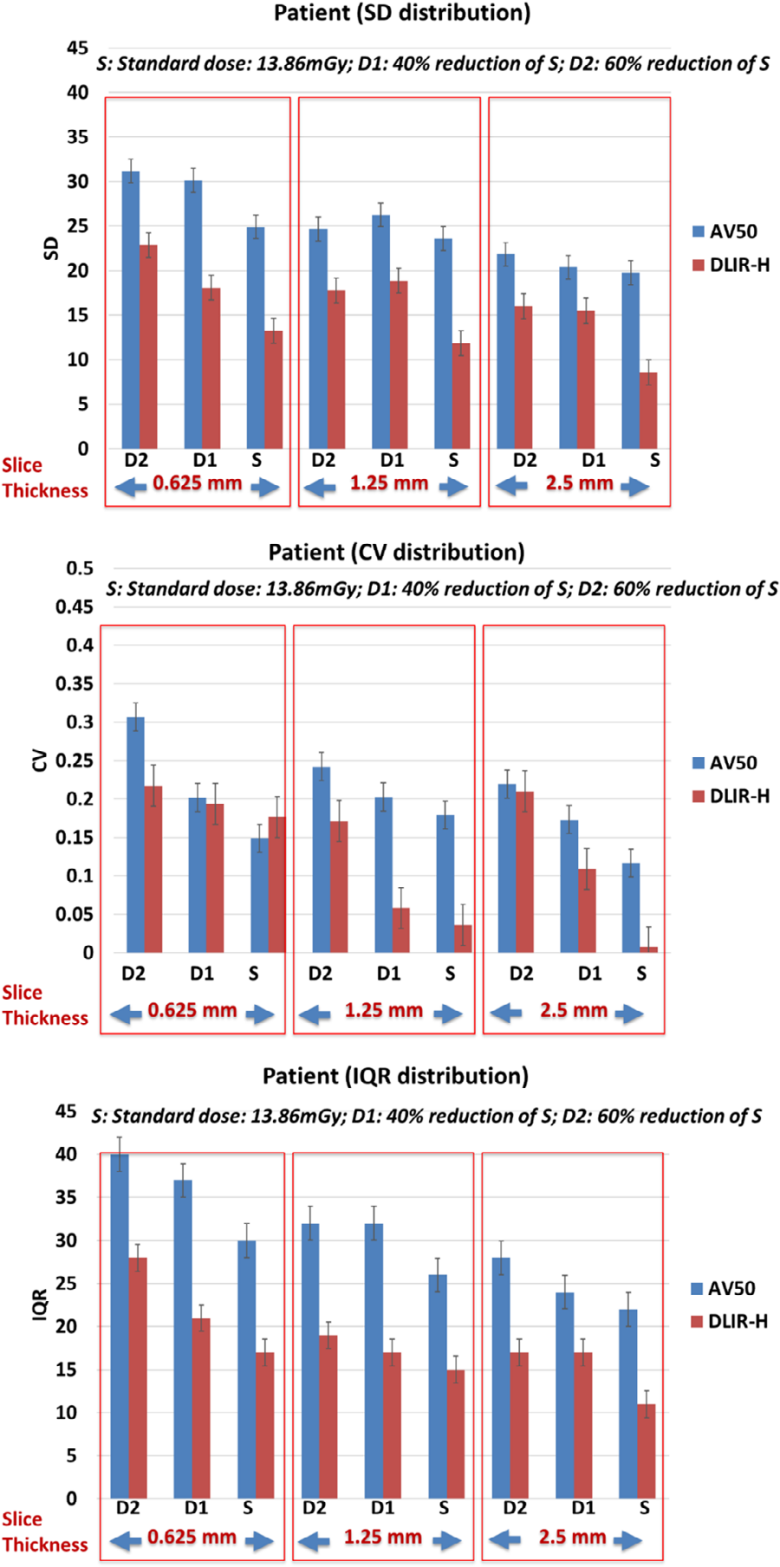
Distribution of SD (top) CV (middle) and IQR (bottom) extracted from the ROIs of an in‐vivo human liver scan, with changes in slice thickness (0.625 mm vs. 1.25 mm vs. 2.5 mm), dose (standard dose (S), 40% of S (D1) and 60% of S (D2)) and reconstruction algorithm (AV50 vs. DLIR‐high).

Effect of slice thickness, dose levels and reconstruction on first order radiomic features: SD, CV and IQR: In general, as previously observed in both phantom, SD and IQR improves as slice thickness increases (coarse *z*‐axis resolution) (Figure 4). These quantities also improve as dose increases (Figure 4). At slice thicknesses of 1.25 mm and 2.5 mm, the relative reduction of SD, CV, and IQR are also higher when using DLIR‐high compared to AV50 (Figure 4). This means that at slice thicknesses of 1.25 mm and 2.5 mm for the same changes in dose, that is, D2 to D1 to S, the associated improvements in SD, CV and IQR are comparatively higher when using DLIR‐high than AV‐50. However, at the lowest resolution of 0.625 mm, this is not observed, possibly due to increased noise associated with small slice thickness. While the improvements of SD and IQR with increasing dose are higher when using DLIR‐high compared to AV50. The improvements of CV with increasing dose are lower when using DLIR‐high compared to AV50. This observation may be due to the non‐linear operations involved in implementing the DLIR‐high reconstruction. All trend tests were statistically significant (*p* < 0.01) The details of slopes and statistical trend tests were shown in Table 2.

### 3.3 Results across all three imaging scenarios

Comparison of the effect of slice thickness, dose levels and reconstruction on first order radiomic features: SD, CV, and IQR: In general, the absolute values for IQR, CV and SD were best for the water phantom compared to the anthropomorphic liver phantom and in‐vivo human liver scans, for all image settings (Figure 2–4). Similar trends of IQR, CV and SD were observed on the liver phantom, the patient liver scan and the large water phantom (Figure 2–4). The absolute values for IQR, CV and SD are comparable between the liver phantom and the patient liver (Figure 2–4). All trend tests were statistically significant (*p* < 0.01) The details of slopes and statistical trend tests were shown in Table 2.

### 3.4 Validation of the reported findings using FBP on the large water phantom scans

Are these observations due to the non‐linear modifications of image by AV50 and DLIR‐high? Changes in CV with reduction in dose and slice thickness were different when using FBP compared to AV50 and DLIR‐high (Figure 2). Higher (worser) values of CV, IQR, and SD were seen in FBP compared to DLIR‐high and AV50 (Figure 2). The trend of reducing SD with increasing dose showed better Poisson characteristics using FBP compared to AV50 and DLIR‐high, that is, the values of CV reduced by half when the radiation dose was increased by four times the original amount.

## 4 DISCUSSION

It is increasingly common for institutions to adopt low dose scans to satisfy the as low as reasonable achievable (ALARA) criteria for in‐vivo radiological imaging. Changes in dose levels can be bought about by varying the tube voltage, tube current and exposure time. Decreasing dose leads to an increase in noise levels which reduces image quality. To try to alleviate this reduction in image quality due to noise, more advanced reconstruction algorithms such as AV50 and DLIR are implemented. Similarly, prior studies have shown quantitative image analyses such as radiomics analysis and segmentation are impacted by different choice in slice thickness and CT dose.^29^ However, a clear consensus has not emerged. The improved image quality by the increase in z‐resolution (thinner slice thickness) may be offset by an increase in sampling noise due to reducing slice thickness, which may affect quantitative measures more drastically than qualitative measures.

Our data suggests that image acquisition parameters relating to dose, reconstruction and slice thickness strongly influence radiomic feature reproducibility; particularly simple first‐order texture metrics such as SD, CV and IQR as reported in this study. The dispersion of values from a mean captured by these 3 metrics can be caused due to imaging related noise or due to underlying internal structures. Ideally, for radiomics to truly serve as reliable diagnostic, prognostic and treatment response assessment technique, the dispersion of values from a mean (radiological texture) must be based on the changes in underlying structures in response to pathology and not imaging variables. Therefore, the establishment of reliable image quality thresholds and reproducibility are critical to differentiate changes due to imaging versus changes in structure, without which radiomics results may be subject to strong acquisition bias and its variability across different centers. By using a structureless CT quantity such as water, as done in our study, the thresholds for performing radiomics, whose underlying goal is to detect pathological differences amidst varying noise characteristics have been established. It is shown that these baselines vary with changes in dose, reconstruction, and slice thickness and that these trends are seen in anthropomorphic liver phantoms and in an in‐vivo scan of the liver in a human patient, with the lowest values of variation (noise) established in water phantom, followed by the anthropomorphic liver phantom and the highest in in‐vivo patient liver scan. The baselines found using the large water phantom are recommended to serve as the minimum threshold for conducting radiomics analysis as well as a meaningful CT QA program. ROIs with SD, CV and IQR variabilities below that of water under the various imaging setting should not be used to conduct radiomics analysis. Also, while the absolute values are different across different settings and phantoms, when comparing the variation of CV, SD and IQR, similar trends were found, which validates that the observed trends are independent of the type of phantom. Also, noting that across all settings the water phantom using FBP showed the worst values of CV, SD and IQR compared to other reconstruction algorithms and phantoms. This further supports the rationale for using a structureless quantify such as water to establish the baseline threshold of imaging noise prior to conducting radiomics.

Based on the evaluation of the effect of slice thickness, dose levels and reconstruction on first order radiomic features: SD, CV and IQR using a large water phantom, the study shows that these quantities improve as slice thickness and dose increases, respectively. In general, an increase in the slice thickness and dose reduces the image noise, contributing to lower values of SD, CV and IQR. This observation is in line with studies that show higher reproducibility of radiomic features with increases in dose. The use of AV50 and DLIR compared to FBP led to blurring which in turn results in the reduction of SD, CV and IQR. However, despite the reduction of noise by the AV50 and DLIR,^11^, ^30^ studies in the literature report fewer reproducible features when compared with FBP (the reference standard).^23^, ^24^ Typically, for a well‐designed CT scanner, image noise (quantum mottle) should be statistical. This means that random variations in detected x‐ray intensity as compared to other sources of noise such as electronic and other sources, should be the main contributor to image noise. Quantitatively, the Poisson distribution is used to characterize the size of random variations, typically as the SD (square root of mean intensity or dose). This means that SD can be reduced by increasing the radiation dose to the slice, for example, by increasing mAs, slice thickness, or other factors. In this study, the Poisson statistics were more stringently noted in FBP than DLIR‐high and AV50, due to the non‐linear nature of the latter two algorithms. AV50 initially uses information obtained from the FBP algorithm to initiate image reconstruction and focuses on noise reduction primarily through statistical approaches.^33^ Specifically, the method modifies pixel by pixel values over multiple iterative processes of converging the FBP pixel values to those predicted by the noise model. While this approach reduces the noise and thus allows reducing the dose, it also modifies the texture of the image.^33^, ^34^, ^35^The posterization effect of using AV50 can hinder interpretation, which limits the use of the high iterative levels to reduce dose in clinical practice. This image modification, despite the reduction in noise perhaps explains the poor concordance observed between features extracted from images with AV50 application compared to FBP. These findings in line with literature reports of studies comparing grey‐level co‐occurrence matrix (GLCM) and intensity histogram (IH) features obtained from FBP and model‐based iterative reconstruction techniques.^36^ By design, the DLIR‐high algorithm was developed to overcome the limitations of IR algorithms and be able to differentiate signal from noise to reduce reconstructed image noise without changing its texture. However, as with all deep learning solutions, its performance will be strongly dependent on the images used to train the model and robustness of the associations learnt. While not as much as AV50, even with DLIR, there is some image blurring.^37^ With the increased use of AV50 and DLIR reconstructions, and lack of consensus on the level of blurring, care should be taken in the extraction of quantitative metrics such as features using heterogeneous data extracted using variable scan protocols.

When using the anthropomorphic liver and in‐vivo liver scans mostly similar trends of SD and IQR as observed in the water phantom were reported using the AV50 and DLIR reconstruction. For the same analysis, the trend followed by CV was different with varying dose particularly when using the lowest slice thickness of 0.625 mm. However, the differences in CV were not significantly different. This deviation in performance of CV was different when using FBP reconstruction. When FBP reconstruction was used, as seen with the other variables, a monotonic decrease of CV was seen with increasing dose and slice thickness. The deviation in CV trend when using AV50 and DLIR may be due to the non‐linear transformations that play an important role while implementing the technique. The differences in the trends of CV, SD and IQR when using different reconstruction algorithms pose a serious challenge when attempting to standardize radiomics for use in multicenter analysis. Linear differences in metrics due to variation in imaging variables are easier to correct/ harmonize compared to non‐linear effects. Therefore, isolating the effects of imaging variables with linear changes to simple first order texture metrics, may serve as a first step towards the effective harmonization of heterogeneously collected radiomics data for large scale multicenter radiomics.

There are several limitations to this study. First, studies evaluating the effects of modulation of dose, slice thickness and reconstruction technique on radiomic metrics require multiple scans; however, our study was limited to phantoms and a single patient. Therefore, we did not test the interaction for assessing the slope difference between settings, for example, whether the slope associated with decreasing dose differs across different slice thickness levels. The water phantom used was circular, filled with liquid water and not representative of complex patient anatomy; however, the goal of using the water phantom was to establish the baselines for SNR thresholds to conduct radiomics analysis. To address this issue of patient anatomy an anthropomorphic phantom was used; however, the phantom texture was uniform and was imaged without other organs and without artifacts observed during patient image acquisition procedures. Therefore, both phantoms lacked the complexity of tissue texture seen in real patient scans, warranting the need for newer phantoms that better capture the tissue properties, particularly multiphase CT. In‐vivo scans from a real patient were added to compare the findings we observed from the other two phantoms and image settings. However, in all cases the level of noise reduction achieved using both AV50 and DLIR was fixed, a sensitivity analysis using different levels of reconstruction blurring was not performed. However, studies from literature show that increased levels of ASIR and DLIR filtering led to increased blurring.^33^, ^34^, ^37^ Therefore, if FBP can be considered as AV0 (zero), we do observe that at AV50, the values of CV SD and IQR are lower than those at AV0 for the same settings. In our study 4 circular ROIs of ∼1 cm diameter each were manually drawn over 5 adjacent CT slices and the edges of the ROI were not included to avoid CT imaging artefacts. Different segmentation strategies employed by various studies can affect the quantification of radiomic features and their reproducibility and we did not comprehensively test this variability. Yet another limitation of this study was that only one type of GE scanners was used, the reproducibility of our findings across other CT vendors were not studied. Such studies are warranted considering the different CT vendors use different dose reduction techniques to make CT safer for use across different populations without compromising image quality. In this study, we also did not consider the differences in performance due to using axial versus helical scanning.

Despite these limitations, these findings underscore the influence of noise on first‐order radiomic metrics and demonstrate the feasibility of using water phantoms as quality assurance standards in radiomics studies, particularly when using heterogenous data from multicenter studies. Midya et al. conducted similar studies with a goal to study the reproducibility of CT radiomic features by varying tube current, noise index, and reconstruction [adaptive statistical iterative reconstruction (ASiR)], parameters that are increasingly varied by institutions seeking to reduce radiation dose in their patients.^17^ These findings are extended in this work by adding to the variables DLIR for reconstruction, slice thickness and variable dose. In this study, a large CT water phantom compared to a small one, which is more suitable for liver imaging applications was used. In future studies, we hope to build texture patterns which are better suited to study higher‐order texture metrics and assess their reproducibility and clinical predictability across different CT vendors and imaging protocols. In addition, oval extensions to circular phantoms will be studied. Despite the difference in volumes due to differences in slice thicknesses, we did not calculate a weighted SD, CV, IQR taking into account the size or volume of each voxel.

This was done because the goal of our study was to choose radiomic metrics as measured by the radiomics software (here CaPTk). And, these three measures, that is, CV, SD and IQR are IBSI compliant, which means that their design and implementation are benchmarked and thus robust. Prior radiomics studies suggest resampling of data prior to running the radiomics software, however, we wanted to assess this difference as well, as many radiomics studies use diverse slice thicknesses. In future studies, we will explore using the weighted SD and CV approach. Also, DLIR‐H is not commonly used as compared to DLIR‐M due to its more aggressive noise suppression and resolution loss. However, in our study the goal was not to compare between different DLIRs (DLIR‐M vs. DLIR‐H) but to compare between AV50 which is an iterative reconstruction method versus DLIR‐H which is a deep learning‐based iterative reconstruction method. In addition, the institution that we partnered with to acquire the phantom images uses DLIR‐H frequently. On a different note, we do agree that noise suppression from ASIR‐V or from DLIR is lesion contrast dependent, especially when the contrast is low. It is expected that the non‐linear aspects may change and complicate the image structure(landscape) and this has been well documented in the literature. Indeed, this is one reason we supply water baths for the readers to relate to radiomics effects in an (almost) “structureless” versus structured CT images. In this way the reader may compare some of the radiomics effects of linear versus nonlinear (iterative) algorithms in structured versus non structured backgrounds. Also, while it will be valuable to provide the quantitative relationship between the first order feature and thickness and dose, this a task best explored using a texture phantom with gradually varying texture values, which a line of research work currently being pursued in our laboratory and others.

In conclusion, the present study demonstrates that not unexpectedly, signal to noise ratio which is an underplay for various factors within the imaging and image processing pipeline plays an important role in quantitative imaging and reproducibility of radiomic features. It is shown that the variation in signal to noise ratio due to the choice of CT reconstruction algorithms, slice thickness and dose is significant and warrants additional studies to create harmonization techniques to reduce this variability prior to use in radiomics studies., particularly for portability in comparison of multicenter radiomics results.

## AUTHOR CONTRIBUTIONS

Bino Varghese—primary author of the paper, idea development, data processing, and analysis; Steven Cen—statistical analysis; Kristin Jensen—imaging protocol development and acquisition of phantom scans; Joshua Levy—idea and phantom development; Hilde Andersen—imaging protocol development; Anselm Schulz—guidance on clinical radiation physics and providing patient scans; Xiaomeng Lei—organized and curated patient data for statistical analysis; Vinay Duddalwar—guidance in the area of radiomics; David Goodenough—overall guidance and provided the clinical insight and context to the study. All the authors reviewed the manuscript and approved the final submission.

## CONFLICT OF INTEREST STATEMENT

David Goodenough is a Consultant to The Phantom Laboratory, Salem, NY. Joshua Levy is an employee and Founder of The Phantom Laboratory, Salem, NY.

## ETHICS STATEMENT

For all procedures performed in studies involving human participants written informed consent was obtained and has secured all the required IRB approvals.
